# Unravelling the Role of the Pentafluoroorthotellurate Group as a Ligand in Nickel Chemistry

**DOI:** 10.1002/chem.202202016

**Published:** 2022-09-08

**Authors:** Alberto Pérez‐Bitrián, Kurt F. Hoffmann, Konstantin B. Krause, Günther Thiele, Christian Limberg, Sebastian Riedel

**Affiliations:** ^1^ Fachbereich Biologie, Chemie, Pharmazie Institut für Chemie und Biochemie – Anorganische Chemie Freie Universität Berlin Fabeckstraße 34/36 14195 Berlin Germany; ^2^ Institut für Chemie Humboldt-Universität zu Berlin Brook-Taylor-Straße 2 12489 Berlin Germany

**Keywords:** coordination chemistry, fluorine chemistry, ligand field theory, nickel, pentafluoroorthotellurate

## Abstract

The pentafluoroorthotellurate group (teflate, OTeF_5_) is able to form species, for which only the fluoride analogues are known. Despite nickel fluorides being widely investigated, nickel teflates have remained elusive for decades. By reaction of [NiCl_4_]^2−^ and neat ClOTeF_5_, we have synthesized the homoleptic [Ni(OTeF_5_)_4_]^2−^ anion, which presents a distorted tetrahedral structure, unlike the polymeric [NiF_4_]^2−^. This high‐spin complex has allowed the study of the electronic properties of the teflate group, which can be classified as a weak/medium‐field ligand, and therefore behaves as the fluoride analogue also in ligand‐field terms. The teflate ligands in [NEt_4_]_2_[Ni(OTeF_5_)_4_] are easily substituted, as shown by the formation of [Ni(NCMe)_6_][OTeF_5_]_2_ by dissolving it in acetonitrile. Nevertheless, careful reactions with other conventional ligands have enabled the crystallization of nickel teflate complexes with different coordination geometries, i.e. [NEt_4_]_2_[*trans‐*Ni(OEt_2_)_2_(OTeF_5_)_4_] or [NEt_4_][Ni(bpyMe_2_)(OTeF_5_)_3_].

## Graphical abstract

The homoleptic [Ni(OTeF_5_)_4_]^2−^ anion has been selectively prepared and arises as an analogue of [NiF_4_]^2−^, but featuring the discrete tetrahedral geometry of the heavier halonickelates. This high‐spin complex has enabled the investigation of the electronic properties of the [OTeF_5_]^−^ group in coordination chemistry, showing that it behaves as the fluoride also in ligand‐field terms.

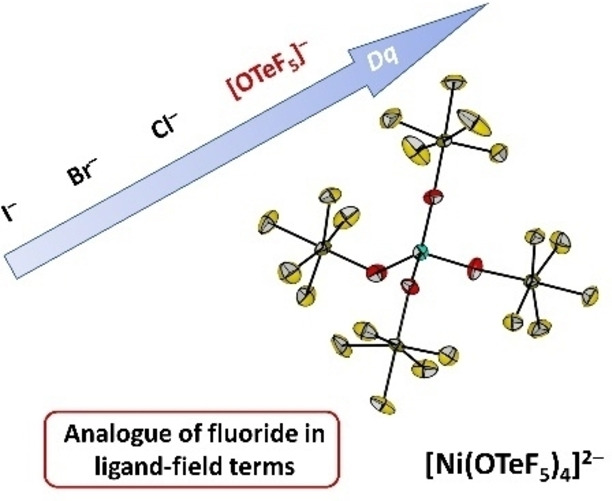

## Introduction

Nickel fluorides and the related fluoronickelates have been known for decades and their chemistry has been thoroughly studied.^[1]^ These species form a very special class of compounds, which significantly differ from the heavier nickel halides in mainly two aspects: firstly, the oxidation state +IV is only accessible with fluorides in the well‐known [NiF_6_]^2−^, as well as in the unstable and strong oxidizer NiF_4_ formed therefrom; secondly, the structures of the different halonickelates in the most common oxidation state +II are different. Whereas the [NiX_4_]^2−^ anions (X=Cl, Br, I) are commonly tetrahedral molecules, [NiF_4_]^2−^ forms layers containing {NiF_6_} octahedra connected by shared corners (i.e. bridging fluorides), therefore adopting a structure comparable to that of SnF_4_.^[2]^ Although more unusual, nickelates containing heavier halides and Ni centers with octahedral coordination are also known, such as [Ni_3_Cl_12_]^2−^.^[3]^

Within this context, one very interesting analogue of the fluoride is the pentafluoroorthotellurate group (teflate, OTeF_5_), since it is also highly electronegative, yet shows a high steric demand.^[4]^ These properties, together with its high charge delocalization and robustness against electrophiles and oxidizers make it a unique ligand, able to stabilize species that are usually the only known analogues of the corresponding fluorides. However, in contrast to the fluoride, it is much less prone to acting as a bridging ligand.[^4^, ^5^] The teflate group has allowed the isolation of a wide range of weakly coordinating anions,^[6]^ for example [Al(OTeF_5_)_4_]^−^,^[7]^ as well as transition‐metal complexes in high oxidation states, as in Mo(OTeF_5_)_6_,^[8]^ and highly reactive species, such as [XeOTeF_5_][Sb(OTeF_5_)_6_].^[9]^ Interestingly, in comparison to the high number of main‐group teflate species, transition‐metal teflate complexes are less abundant, and are mainly known with early transition metals.[^4^, ^5^]

In the particular case of nickel, the coordination of the teflate ligand has remained elusive for decades. The reaction of [NiCl_2_(dppe)] (dppe=1,2‐bis(diphenylphosphino)ethane) and [Ag(OTeF_5_)(toluene)_2_] only led to metallic deposits, similarly to other phosphine complexes of group 10 metals.^[10]^ This behavior was attributed to the redox incompatibility between the teflate and the phosphine ligands, which was disproved by the synthesis of [Pt(OTeF_5_)_2_(PEt_3_)_2_].^[11]^ The homoleptic anion [Ni(OTeF_5_)_4_]^2−^ was mentioned in a review in 1993 as a potential weakly coordinating anion, yet no experimental details were provided, not even years later.^[12]^ In fact, it was not until 2018 that the first and only nickel teflate complexes were reported by our group, i.e. [Ni(OTeF_5_)_2_(Hacac)_2_] and *cis‐*[Ni(^*i*Pr^Im)_2_(OTeF_5_)_2_] (acac=acetylacetonate, ^*i*Pr^Im=1,3‐bis(isopropyl)imidazolin‐2‐ylidene).^[13]^

Since the Pd(II) analogue [Pd(OTeF_5_)_4_]^2−^ is known,[^14^, ^15^] and other homoleptic {NiO_4_} anions have been also prepared,[^16^, ^17^, ^18^] we envisioned that salts of the [Ni(OTeF_5_)_4_]^2−^ anion should be also stable. Herein we present the synthesis, characterization and study of the hitherto unknown [Ni(OTeF_5_)_4_]^2−^ anion, which has been prepared by using neat ClOTeF_5_ as a teflate‐transfer reagent. The extensive use of Ni(II) species to study fundamental properties in coordination chemistry made this compound especially suited to undertake an unprecedented study of the ligand‐field properties of the teflate group. The OTeF_5_ ligands in this complex can be easily substituted, but a careful choice of ligands and reaction conditions leads to nickel teflate complexes with different coordination geometries.

## Results and Discussion

### Synthesis and characterization of [NEt_4_]_2_[Ni(OTeF_5_)_4_]

Various attempts to prepare salts of the [Ni(OTeF_5_)_4_]^2−^ anion by modifying previous synthetic procedures of related species were unsuccessful. Contrary to the usefulness of PdCl_2_ as a starting material for the synthesis of the analogous [Pd(OTeF_5_)_4_]^2−^ anion,[^14^, ^15^] NiCl_2_ proved to be unreactive. Considering the low solubility of this compound, we tried the reaction of different [NiX_4_]^2−^ salts (X=Cl, Br, I) with AgOTeF_5_, but only incomplete substitutions and mixtures of species were obtained. Similar results were found when using [NiBr_2_(dme)] (dme=1,2‐dimethoxyethane) and [NMe_4_][OTeF_5_] or AgOTeF_5_ as starting materials, unlike in case of the recently reported synthesis of [Ni(OC_4_F_9_)_4_]^2−^ using Na[OC_4_F_9_].^[16]^ Although the use of nickelocene proved to be useful for the synthesis of [Ni(OR)_4_]^2−^ anions (R=C_6_F_5_, C_6_H_3_(CF_3_)_2_, C_4_F_9_),^[18]^ in our case the reaction of nickelocene, HOTeF_5_ and [NMe_4_][OTeF_5_] only led to mixtures of unidentified products which could not be separated.

**Scheme 1:**
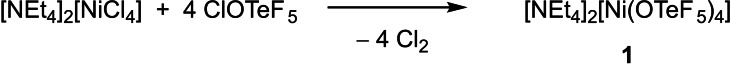
Synthesis of [NEt_4_]_2_[Ni(OTeF_5_)_4_] (**1**).

Given the low solubility of Ni halides and the low coordinating ability of the teflate ligand, we envisioned a solvent‐free synthesis of the [Ni(OTeF_5_)_4_]^2−^ anion by using neat ClOTeF_5_ as the teflate‐transfer reagent.^[19]^ The reaction of [NEt_4_]_2_[NiCl_4_] with an excess of ClOTeF_5_ leads to the release of Cl_2_ and concomitant formation of a dark brown slurry, which upon removal of all volatiles under dynamic vacuum resulted in a dark blue powder (Scheme 1). The transformation takes place quantitatively to yield [NEt_4_]_2_[Ni(OTeF_5_)_4_] (**1**) as a pure substance, as demonstrated by mass balance and elemental analysis. On the other hand, the formation of chlorine was proved by gas‐phase UV‐Vis spectroscopy (see Figure S1). The isolated compound is extremely moisture‐sensitive, but under strict inert conditions, it is thermally stable and decomposes only above 220 °C, as determined by thermogravimetric analysis (see Figure S10). Interestingly, the analogous reaction between neat ClOTeF_5_ and NiCl_2_ does not take place, probably due to the high stability of the highly ionic nickel species. This behavior is in contrast with that of FeCl_3_, which is known to react with ClOTeF_5_ in SO_2_ClF or C_4_F_9_SO_2_F to yield Fe(OTeF_5_)_3_.^[20]^

Contrary to HOTeF_5_, AgOTeF_5_ or B(OTeF_5_)_3_, which have been extensively used in the synthesis of teflate derivatives,[^4^, ^5^] the hypochlorite ClOTeF_5_ has resulted to be of limited use and only a few derivatives have been prepared therefrom.[^8^, ^20^, ^21^] In our case, the use of the neat substance and the need of no solvent is the key factor leading to a successful reaction for two reasons. Firstly, it allows to circumvent the need of easily accessible and soluble nickel precursors, the latter being an important drawback for nickel halides. Secondly, the resulting compound is unstable in donor solvents such as MeCN (see below).

**Figure 1:**
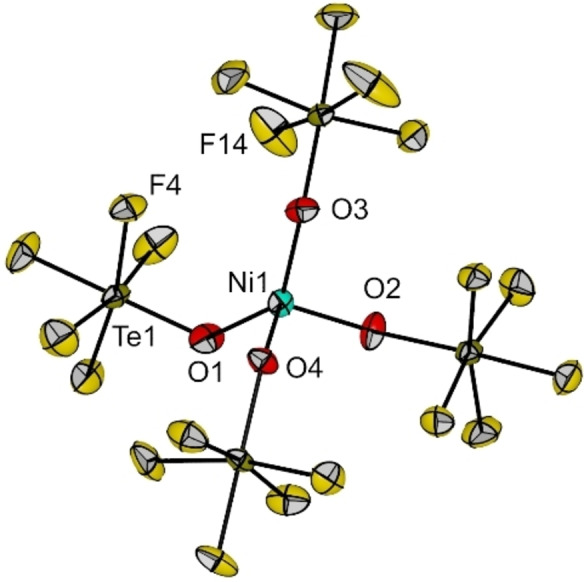
Molecular structure of the [Ni(OTeF_5_)_4_]^2−^ anion in the solid state as found in crystals of **1**. The [NEt_4_]^+^ cations have been omitted for clarity. Displacement ellipsoids set at 50 % probability. Selected bond lengths [pm] and angles [°]: Ni1−O1 195.2(7), Ni1−O2 194.2(7), Ni1−O3 195.5(7), Ni1−O4 193.2(6), O1−Ni1−O2 96.6(3), O1−Ni1−O3 138.4(3), O1−Ni1−O4 107.8(3), O2−Ni1−O3 108.7(3), O2−Ni1−O4 115.3(3), O3−Ni1−O4 90.9(3). For crystallographic details see Supporting Information.

Crystals of [NEt_4_]_2_[Ni(OTeF_5_)_4_] (**1**) suitable for X‐ray diffraction were grown by slow evaporation of a saturated solution of the compound in *ortho‐*difluorobenzene (*o*DFB) at room temperature. Compound **1** crystallizes in the monoclinic space group *P*2_1_/*c*. (Figure 1). The coordination geometry of the [Ni(OTeF_5_)_4_]^2−^ anion can be described as a distorted tetrahedron, as becomes clear from the O−Ni−O angles, which range between 90.9(3) and 138.4(3)°. The deviation from the 109.5° in a regular tetrahedron becomes much more prominent than in the related salt [NEt_4_]_2_[NiCl_4_], where angles are in the narrow range 106.83(14)–110.81(7)°.^[22]^ To describe the distortions found in four‐coordinate complexes in a more objective way, the geometry parameter *τ*_4_ was developed by Houser et al.^[23]^ A perfect tetrahedral geometry would present a value of *τ*_4_=1, which is almost the case for the [NiCl_4_]^2−^ anion (0.98). In our case, the *τ*_4_ parameter for the [Ni(OTeF_5_)_4_]^2−^ anion has a value of 0.75, which shows clearly the distortion. The distortion of compound **1** is most probably due to electronic rather than to steric reasons, as the related [NEt_4_][Al(OTeF_5_)_4_] salt, which shows much shorter Al−O bond lengths (175.0(14)–178.8(13) pm), is less distorted.^[24]^ In fact, a careful analysis of the Ni⋅⋅⋅F intramolecular interactions in the solid state shows that the distances Ni1−F4 (321.6(7) pm) and Ni−F14 (321.3(7) pm) are much shorter than the average Ni⋅⋅⋅F contacts. These interactions might have a key role in the distorsion of the ideal tetrahedral geometry found in the [Ni(OTeF_5_)_4_]^2−^ anion, as the largest O1−Ni1−O3 angle (138.4(3)°) is the one that involves the teflate ligands to which F4 and F14 belong (see Figure 1). Interestingly, only one additional tetrahedrally coordinated teflate dianion complex has been characterized by single‐crystal X‐ray diffraction, i.e. [Hg(OTeF_5_)_4_]^2−^, which also shows a significant distortion (bond angles ranging from 86.3(2) to 125.9(3)°).^[25]^

Interestingly, this is one of the few reported crystal structures of a Ni(II) compound with a {NiO_4_} coordination, which contains only monodentate ligands.[^17^, ^18^] Additionally, it is the only one in which the oxygen atoms do not establish strong interactions with a metal cation, as observed, for instance, in {K(18C6)}_2_[Ni(OC_6_F_5_)_4_]^[18]^ or in [Na(dme)]_2_[Ni(OC(CF_3_)_3_)_4_].^[16]^ It is also interesting to note, that in the structure of the palladium analogue, which shows a planar {PdO_4_} coordination, the O atoms of the teflate ligands are bonded to Ag(I) ions.[^14^, ^15^] The Ni−O bond lengths, however, are in the range of other tetrahedral Ni(II) complexes with a {NiO_4_} environment.^[18]^ Interestingly, all halide complexes [NiX_4_]^2−^ (X=Cl, Br, I) show a tetrahedral geometry except [NiF_4_]^2−^, which shows an extended structure consisting of layers of corner‐sharing {NiF_6_} octahedral units in K_2_[NiF_4_].^[26]^ Therefore, [NEt_4_]_2_[Ni(OTeF_5_)_4_] (**1**) arises as an analogue of [NiF_4_]^2−^ with a discrete structure instead of a polymeric one.

The IR spectrum of [NEt_4_]_2_[Ni(OTeF_5_)_4_] shows the two most characteristic stretching vibrations of the teflate ligand (see Figure S3): the Te−F vibration appears at 662 cm^−1^, whereas the Te−O one at 834 cm^−1^. As the latter appears above 820 cm^−1^, the Ni−O bonds can be considered to have an ionic character,^[27]^ although it is far away from the highest reported Te−O vibration, i.e. 873 cm^−1^ in Cs[OTeF_5_],^[28]^ or 867 cm^−1^ in [N^*n*^Bu_4_][OTeF_5_], where the teflate group seems not to be so affected by the positive charge.^[29]^

**Figure 2:**
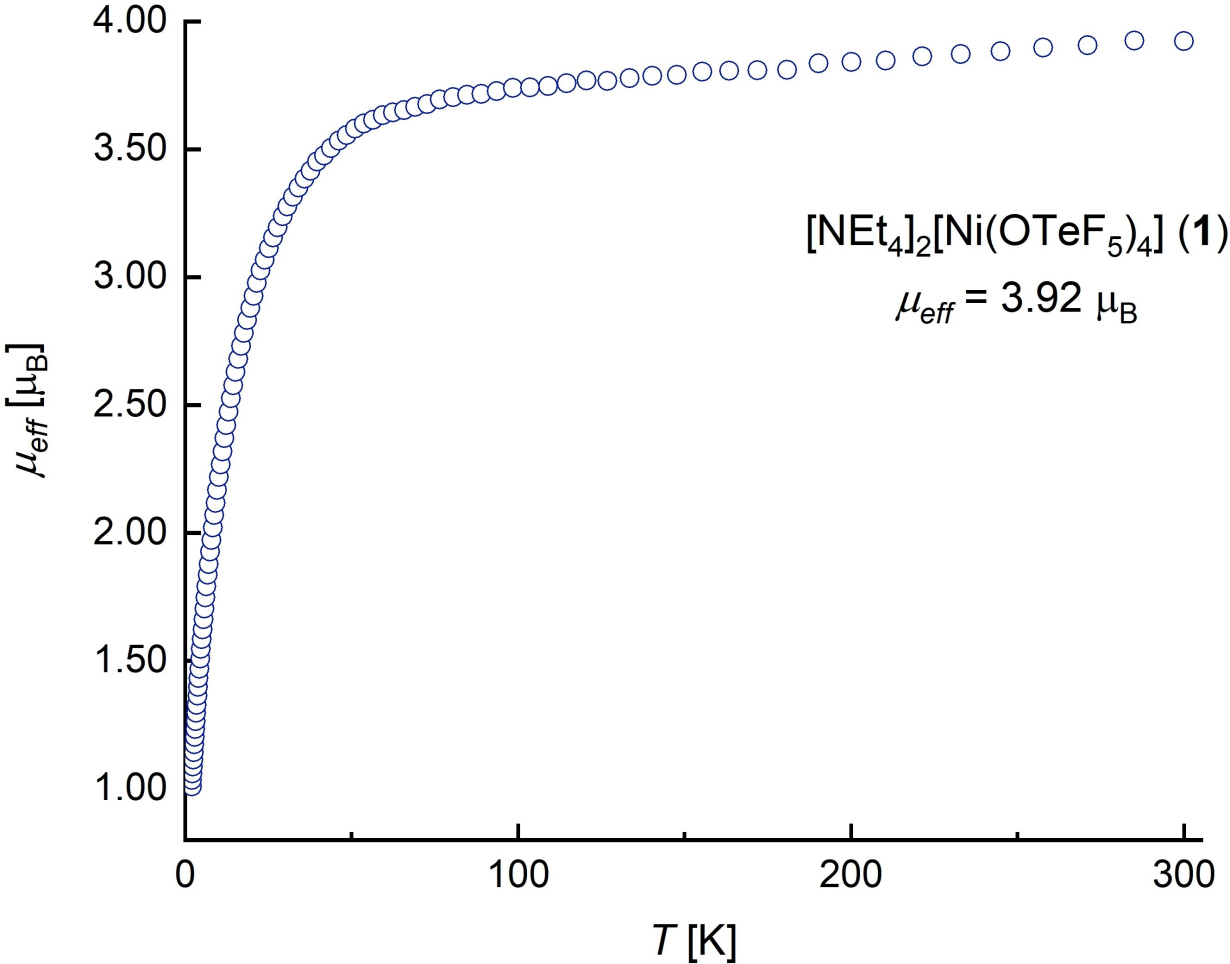
*μ_eff_* versus *T* plot for compound [NEt_4_]_2_[Ni(OTeF_5_)_4_] (**1**), obtained with a SQUID magnetometer.

For tetrahedral complexes such as **1**, typically high‐spin configurations are observed.^[30]^ In the case of a d^8^ system as it is the case in [Ni(OTeF_5_)_4_]^2−^, this leads to a *S*=1 state, which implies the existence of two unpaired electrons. Accordingly, only two broad signals of very poor quality due a bad signal‐to‐noise ratio appear in the ^19^F NMR in CD_2_Cl_2_, which is in line with the coordination of the teflate ligands to a paramagnetic Ni(II) center. Therefore, we decided to investigate the magnetism of [NEt_4_]_2_[Ni(OTeF_5_)_4_] (**1**). We used two different techniques to determine the effective magnetic moment of **1** at room temperature (see Supporting Information for details). By using the magnetic susceptibility balance, the effective magnetic moment was determined to be *μ_eff_* =3.62 μ_B_, whereas with a SQUID magnometer a value of *μ_eff_* =3.92 μ_B_ was obtained. The latter was found, as expected, to be independent of the temperature (Figure 2). These values are somewhat higher than the spin‐only value of *μ_eff_* =2.83 μ_B_, calculated for two unpaired electrons. This deviation can be rationalized by orbital contributions due to the tetrahedral structure and low ligand field strength. The observed value of the effective magnetic moment, according to the approximations provided by Figgis,^[31]^ lies in the range of values expected for tetrahedral nickel(II) complexes with weak‐field and medium‐field ligands and hence it classifies the pentafluoroorthotellurate as belonging to this ligand class. In fact, the [NiX_4_]^2−^ anions (X=Cl, Br, I) also show effective magnetic moments in the range of 3.5−3.9 μ_B_.^[32]^ Additionally, other fluorinated alkoxide and aryloxide ligands provide values in the lower part of the range, and were therefore classified also as medium‐field ligands.^[18]^

### Ligand‐field properties of the OTeF_5_ group

Electronic spectroscopy of d metal complexes is useful for the study of structural and bonding properties of coordination compounds.^[33]^ Nickel(II) complexes have been widely used to study the electronic properties of d^8^ ions, which can exist in different coordination geometries.^[34]^ Nevertheless, in the case of tetrahedral complexes, the combination of Ni^II^ with fluoride ligands could not be studied, as the homoleptic tetrafluoridonickelate(II) aggregates to yield higher coordination numbers (see above). The teflate group is known to have comparable electron‐withdrawing properties to the fluoride, as stated in the introduction, and surprisingly, its electronic properties in coordination chemistry have not been reported thus far. The [Ni(OTeF_5_)_4_]^2−^ anion arises then as a suitable option to undertake such study due to its discrete structure.

**Figure 3:**
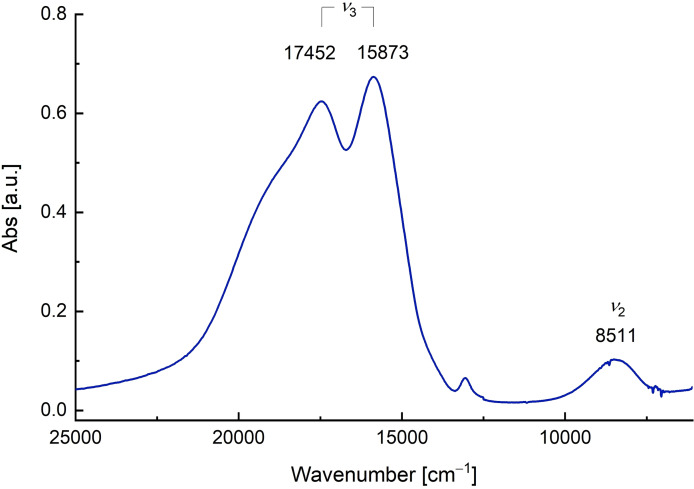
UV‐Vis‐near‐IR spectrum of [NEt_4_]_2_[Ni(OTeF_5_)_4_] (**1**) in CH_2_Cl_2_ (∼0.01 M).

The UV‐Vis‐near‐IR spectrum of [NEt_4_]_2_[Ni(OTeF_5_)_4_] (∼0.01 M in CH_2_Cl_2_) is shown in Figure 3. The two important main bands *ν*_2_ and *ν*_3_ are observed. The former appears at 8511 cm^−1^, whereas the latter consists of two components at 17452 cm^−1^ and 15873 cm^−1^. From them, the values of the ligand‐field parameter *Dq* and the Racah parameter *B* can be calculated according to the equations published by Dou (see Supporting Information for details).^[35]^ In our tetrahedral d^8^ species, which has a T_1_ ground state, these parameters are calculated to be *Dq*=461 cm^−1^ and *B*=896 cm^−1^, respectively.

The spectrochemical series empirically ranks the ligands according to the intensity of the crystal field they create. In terms of the ligand‐field theory, the teflate ligand also behaves similarly to fluoride, as it leads to a greater ligand‐field splitting *Dq* than the chloride in the analogous [NiCl_4_]^2−^. Additionally, with its strong electron‐withdrawing character, it produces a similar energy difference between the d orbitals, which is comparable to other fluorinated ligands, such as [OC_4_F_9_]^−^ or [OC_6_F_5_]^−^ (Table 1).^[18]^ The teflate can be included in the spectrochemical series in a similar position as the fluoride, therefore both being comparably strong ligands in ligand‐field terms:

$$I{}^{-}∼\mathrm{Br}{}^{-}<\mathrm{Cl}{}^{-}<\left[\mathrm{OC}{}_{4}F{}_{9}\right]{}^{-}<\left[OTeF{}_{5}\right]{}^{-}<\left[\mathrm{OC}{}_{6}F{}_{5}\right]{}^{-}<\left[\mathrm{NCO}\right]{}^{-}<\left[\mathrm{OC}{}_{6}H{}_{3}\left(\mathrm{CF}{}_{3}\right){}_{2}\right]{}^{-}$$

**Table 1:** Electronic spectral data and ligand‐field parameters of the [Ni(OTeF_5_)_4_]^2−^ anion and related species.^[a]^

Compound	*ν*_2_ [cm^−1^]	*ν*_3_ [cm^−1^]^[b]^	*Dq* [cm^−1^]	*B* [cm^−1^]	Conditions
[Ni(OTeF_5_)_4_]^2−^	8511	15873, 17452	461	896	CH_2_Cl_2_ (∼0.01 M)
[NiCl_4_]^2−^	7549	14250, 15240	409	795	MeCN (0.01 M)[^34^, ^36^]
[NiBr_4_]^2−^	6995	13230, 14140	379	738	MeCN (0.01 M)[^34^, ^36^]
[NiI_4_]^2−^	7042	14030	382	760	MeNO_2_ (0.01 M)[^34^, ^36^]
[Ni(NCO)_4_]^2−^	9460	15600, 16800	511	841	MeNO_2_ (0.036 M)[^34^, ^37^]
[Ni(OC_4_F_9_)_4_]^2−^	7840	19300	427	1096	CH_2_Cl_2_ ^[18]^
[Ni(OC_6_F_5_)_4_]^2−^	9290	16660	502	877	CH_2_Cl_2_ ^[18]^
[Ni(OC_6_H_3_(CF_3_)_2_)_4_]^2−^	10000	16820	540	868	CH_2_Cl_2_ ^[18]^

[a] All values of *Dq* and *B* have been calculated for this work according to the equations published by Dou,^[35]^ by using the values of *ν*_2_ and *ν*_3_ which are provided in the corresponding entry (see Supporting Information for details). [b] *ν*_3_ was estimated to be the average of the two components observed for such absorption, when this was the case.

According to its location in the spectrochemical series, it can be classified as a weak/medium‐field ligand, as was also concluded from the determined effective magnetic moment of the [Ni(OTeF_5_)_4_]^2−^ anion (see above).

Furthermore, the Racah parameter *B* indicates the repulsion of d electrons in a metal.^[33]^ In comparison to the gaseous free ion, this parameter decreases upon coordination of ligands, indicating a decrease in the interelectronic repulsion in complexes. This reduction of the *B* parameter is the so‐called nephelauxetic effect, which depends both on the metal and the ligands.^[38]^ For a given metal, within the series of halides, the fluoride ligand usually leads to the least repulsion between d electrons, which implies that the *B* parameter associated with it is the largest of all of them. Also the teflate ligand shows a *B* parameter, which is larger than the parameters of the heavier halides (Table 1). However, it is actually similar to those of related O‐donor ligands, which is in agreement with the higher electronegativity of oxygen (in comparison with chloride and the heavier halides), therefore reducing further the interelectronic repulsion of the d electrons.^[33]^ This allows to include the teflate group in the nephelauxetic series of ligands, in order of decreasing *B* parameter (and increasing nephelauxetic effect), as follows:

$$\left[\mathrm{OC}{}_{4}F{}_{9}\right]{}^{-}<\left[OTeF{}_{5}\right]{}^{-}<\left[\mathrm{OC}{}_{6}F{}_{5}\right]{}^{-}∼\left[\mathrm{OC}{}_{6}H{}_{3}\left(\mathrm{CF}{}_{3}\right){}_{2}\right]{}^{-}<\left[\mathrm{NCO}\right]{}^{-}<\mathrm{Cl}{}^{-}<I{}^{-}∼\mathrm{Br}{}^{-}$$

### Chemical behavior of [NEt_4_]_2_[Ni(OTeF_5_)_4_]

**Scheme 2:**
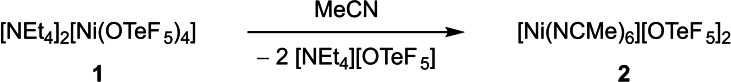
Dissociation of the [OTeF_5_]^−^ in complex **1** upon dissolving in acetonitrile to yield [Ni(NCMe)_6_][OTeF_5_]_2_ (**2**).

As commented previously, compound [NEt_4_]_2_[Ni(OTeF_5_)_4_] (**1**) is very sensitive to humidity, which gives clear evidence of the high lability of the Ni−OTeF_5_ bonds. In fact, when the compound is dissolved in a coordinating solvent, the teflate ligands are rapidly and completely dissociated, whereas the Ni(II) center becomes solvated. This shows that the [OTeF_5_]^−^ group behaves as a weakly coordinating ligand. For instance, upon addition of acetonitrile, the characteristic deep blue color of [NEt_4_]_2_[Ni(OTeF_5_)_4_] (**1**) disappears, giving rise to the typical pale blue color of the octahedrally‐coordinated [Ni(NCMe)_6_]^2+^ (Scheme 2). In fact, the solution of compound **1** in CD_3_CN allows to observe the signals corresponding to the free [OTeF_5_]^−^ anion in the ^19^F NMR spectrum,^[29]^ showing the AB_4_ pattern typical for OTeF_5_ groups, but with a rather poor quality due to the paramagnetism of the cation (see Figure S2). A similar behavior was reported for the complex [Ni(OTeF_5_)_2_(Hacac)_2_] in acetonitrile,^[13]^ as well as for the neutral Ni[EF_6_]_2_ species (E=Bi, Sb),^[39]^ which form the corresponding [Ni(NCMe)_6_][EF_6_] salts upon dissolving in acetonitrile. In related nickel pentafluorooxosulfate compounds, the OSF_5_ ligand was also suggested to undergo dissociation upon dissolving in acetonitrile.^[40]^

**Figure 4:**
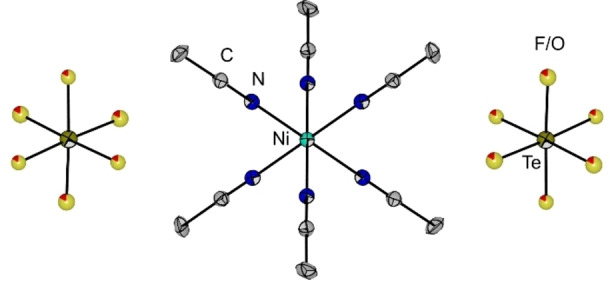
Molecular structure of [Ni(NCMe)_6_][OTeF_5_]_2_ (**2**). Hydrogen atoms have been omitted for clarity. Displacement ellipsoids set at 50 % probability. The F/O disorder is shown by mixed color sites. Selected bond lengths [pm] and angles [°]: Ni−N 206.2(4), N−C 112.9(6), C−C 146.1(7), Te−F/O 179.9(6)/184.3(6) (bond lengths corresponding to *trans‐*standing bonds), N−Ni−N 88.75(15)/91.25(15), Ni−N−C 176.5(4), N−C−C 179.0(6), F/O−Te−F/O 95.7(4)/87.3(4)/84.2(4). For crystallographic details see Supporting Information.

Slow gas diffusion of Et_2_O into an acetonitrile solution of compound **1** at low temperature allowed to obtain purple single crystals of [Ni(NCMe)_6_][OTeF_5_]_2_ (**2**), which proves the described behavior (see Figure 4). The IR spectrum of **2** (see Figure S4) cleanly shows the bands corresponding to the [Ni(NCMe)_6_]^2+^ cation^[39]^ and to the [OTeF_5_]^−^ anion.^[29]^

This salt crystallizes in the trigonal space group *R*‐3. Both the cation and the anions have octahedral geometry. When focusing on the [Ni(NCMe)_6_]^2+^ cation, it can be noticed that it is a distorted octahedron, with similar structural parameters as those found in the related [Ni(NCMe)_6_][SbF_6_]_2_.^[41]^ By its part, there is an inversion twin with symmetry‐related disorder of the oxygen/fluorine atoms in the [OTeF_5_]^−^ group. This F/O disorder was expected, as there is no cation‐anion interaction that can help orient the oxygen atom and overcome such disorder, as was observed in the crystal structure of [(PS)H][OTeF_5_] (PS=1,8‐bis(dimethylamino)naphthalene).^[42]^ In our case, two different Te−F/O bond lengths are observed, i.e. 179.9(6) and 184.3(6) pm, but within the range expected for the free teflate anion, for which the Te−O bond length is 180.3 pm and the average Te−F distance is 187.0 pm.^[42]^ Interestingly, in the related [OSF_5_]^−^ anion, the S−O and S−F bonds can be easily differentiated in the crystal structure of [Cu(NCMe)_4_][OSF_5_], but in this case many H−F and H−O contacts are established between the [OSF_5_]^−^ anion not only with the coordinated MeCN ligands, but also with free acetonitrile molecules.^[40]^

**Figure 5:**
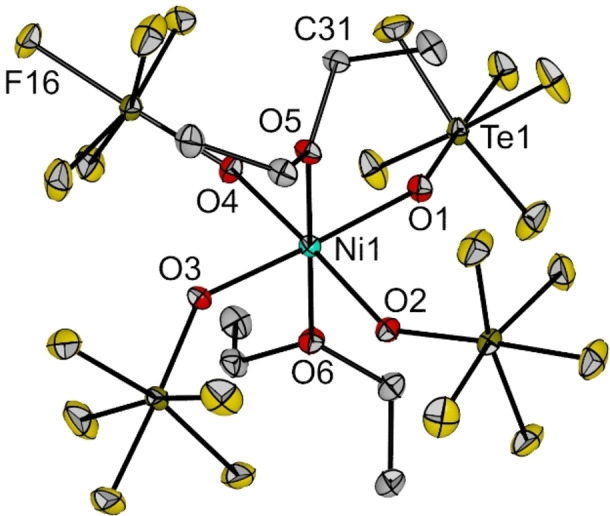
Molecular structure of the [*trans‐*Ni(OEt_2_)_2_(OTeF_5_)_4_]^2−^ anion in the solid state as found in crystals of **3**. The [NEt_4_]^+^ cations and hydrogen atoms have been omitted for clarity, as well as a co‐crystallized CH_2_Cl_2_ molecule. Displacement ellipsoids set at 50 % probability. Selected bond lengths [pm] and angles [°]: Ni1−O1 202.87(16), Ni1−O2 205.92(16), Ni1−O3 202.71(15), Ni1−O4 204.84(16), Ni1−O5 207.61(16), Ni1−O6 208.46(16), O1−Ni1−O2 90.28(6), O2−Ni1−O3 90.39(6), O3−Ni1−O4 88.97(6), O4−Ni1−O1 90.54(6), O1−Ni1−O5 89.63(6), O2−Ni1−O5 92.14(6), O3−Ni1−O5 87.43(6), O4−Ni1−O5 91.39(6), O5−Ni1−O6 179.71(6). For crystallographic details see Supporting Information.

Remarkably, when certain donor solvents enter in contact at low temperature with compound [NEt_4_]_2_[Ni(OTeF_5_)_4_] (**1**), the complete substitution of the teflate ligands can be avoided. This was the case when trying to crystallize compound [NEt_4_]_2_[Ni(OTeF_5_)_4_] (**1**) by vapor diffusion of Et_2_O into a solution of the compound in CH_2_Cl_2_ at −24 °C. In fact, the complex anion is able to coordinate two molecules of diethyl ether in the axial positions of an octahedron, therefore forcing the teflate ligands into the equatorial plane, giving rise to [NEt_4_]_2_[*trans‐*Ni(OEt_2_)_2_(OTeF_5_)_4_]⋅CH_2_Cl_2_ (**3**) (see Figure 5).

Compound **3** crystallizes in the orthorhombic space group *Pcca*. The nickel center in the [*trans‐*Ni(OEt_2_)_2_(OTeF_5_)_4_]^2−^ anion shows a distorted octahedral coordination environment, where the Ni−O bonds to the Et_2_O molecules coordinated in the axial positions are elongated with respect to those corresponding to the teflate ligands (204.09(16) pm (av.) vs. 208.04(16) pm (av.)). This evidences that the Et_2_O ligands are more weakly coordinated to the nickel center than the teflate ligands. Additionally, it is interesting to note that the Ni−O distances in the four teflate ligands in the equatorial plane are longer than in the parent [Ni(OTeF_5_)_4_]^2−^ anion: 204.09(16) pm (av.) vs. 194.5(7) pm (av.) (cf. Figure 1). A similar effect is observed when comparing the Al−OTeF_5_ bond lengths in [PPh_4_][Al(OTeF_5_)_4_] (173.4(2) pm, av.)^[7]^ and in [Li(thf)_4_][*trans‐*Al(OTeF_5_)_4_(thf)_2_] (185.1(6)–186.1(6) pm).^[43]^

These results led us to envision a possible partial substitution of the teflate ligands to form heteroleptic nickel teflate complexes with ancillary ligands. With this aim, we performed the equimolar reaction of compound [NEt_4_]_2_[Ni(OTeF_5_)_4_] (**1**) with 2,2’‐bipyridine (bpy) under different reaction conditions. In none of the cases could we obtain anything different than mixtures of species which could not be separated, but which included the [Ni(bpy)_3_]^2+^ cation complex. This would imply again the easiness of dissociation of the weakly coordinated teflate groups, even when there is not even an excess of the introduced ligand. Similarly, when refluxing NiF_2_ ⋅ 4H_2_O with bpy in methanol, the same cation [Ni(bpy)_3_]^2+^ seems to be formed, whereas the fluorides act as counterions.^[44]^ In contrast, the reaction of the analogue fluoride species Li_2_[NiF_4_] with an excess of pyridine (py) was reported to render crystals of the species [*trans‐*NiF_2_(py)_4_].^[45]^

**Scheme 3:**
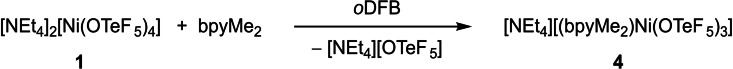
Reaction of complex **1** with bpyMe_2_ to yield [NEt_4_][Ni(bpyMe_2_)(OTeF_5_)_3_] (**4**).

Trying to overcome the easy dissociation, we decided to try the equimolar reaction of compound [NEt_4_]_2_[Ni(OTeF_5_)_4_] (**1**) with 2,2’‐dimethyl‐6,6’‐bipyridine (bpyMe_2_) in *o*DFB. In this case, only one teflate ligand was dissociated and the coordination sphere of the Ni(II) center was extended to form compound [NEt_4_][Ni(bpyMe_2_)(OTeF_5_)_3_] (**4**) selectively (Scheme 3). The methyl groups in the ancillary ligand seem to play here a key role to prevent the dissociation of further teflate ligands. The same ligand was also successful in preventing mixtures of species in the case of compound [Na(dme)_2_][Ni(OC_4_F_9_)_4_], but in this case two [OC_4_F_9_]^−^ anions were dissociated to form the distorted tetrahedral complex [Ni(bpyMe_2_)(OC_4_F_9_)_2_].^[16]^

**Figure 6:**
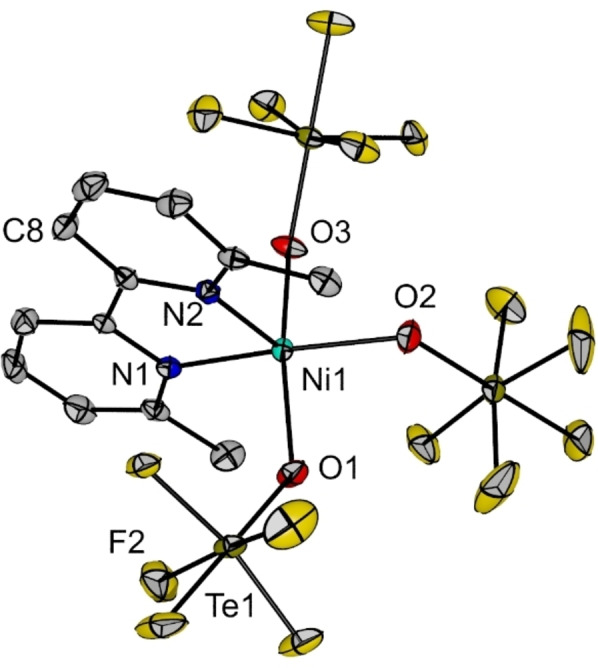
Molecular structure of the [Ni(bpyMe_2_)(OTeF_5_)_3_]^−^ anion in the solid state as found in crystals of **4**. Only one of the two indentendent anions found is shown. The [NEt_4_]^+^ cation and hydrogen atoms have been omitted for clarity. Displacement ellipsoids set at 50 % probability. Selected bond lengths [pm] and angles [°]: Ni1−O1 201.3(4), Ni1−O2 199.7(4), Ni1−O3 206.3(4), Ni1−N1 201.5(5), Ni1−N2 202.1(4), O1−Ni1−O2 88.42(18), O1−Ni1−O3 158.33(14), O2−Ni1−O3 88.47(18), N1−Ni1−N2 82.05(17), O1−Ni1−N2 104.92(16), O2−Ni1−N2 112.48(17), O3−Ni1−N2 96.07(16). For crystallographic details see Supporting Information.

Single crystals of **4** were grown by slow vapor diffusion of *n‐*pentane into a solution of compound **4** in *o*DFB. The species crystallizes in the triclinic space group *P*‐1 and two independent [Ni(bpyMe_2_)(OTeF_5_)_3_]^−^ anions are found in the asymmetric unit, which show no interaction with the cations. In Figure 6 one of the two anions is depicted. This anionic complex shows a fivefold coordinated nickel(II) center with a distorted square pyramidal structure, the structural parameter being *τ*_5_=0.1.^[46]^ The N2 atom occupies the apical position, whereas N1 and the three teflate ligands occupy the four positions of the base. Interestingly, although both Ni−N bond lengths are indistinguishable within the experimental error, the three Ni−O bonds are different, but in all cases elongated with respect to those found in the parent compound **1**.

## Conclusion

The reaction of neat ClOTeF_5_ with [NEt_4_]_2_[NiCl_4_] has led to the synthesis of compound [NEt_4_]_2_[Ni(OTeF_5_)_4_] (**1**), which is extremely moisture sensitive, but thermally stable under inert conditions. Magnetic studies have revealed an effective magnetic moment that indicates two unpaired electrons in the nickel center, therefore accounting for its paramagnetic behavior. The [Ni(OTeF_5_)_4_]^2−^ anion presents a distorted tetrahedral structure, unlike the polymeric [NiF_4_]^2−^, therefore being an analogue of this fluoronickelate, but featuring the discrete structure of the heavier halonickelates. This complex has enabled the unprecedented study of the electronic properties of the teflate group in coordination chemistry, for which values of *Dq*=461 cm^−1^ and *B*=896 cm^−1^ were derived from its UV‐Vis‐near‐IR spectrum. These parameters, together with the value of the effective magnetic moment, demonstrate that the [OTeF_5_]^−^ is a weak/medium‐field ligand, similarly to the fluoride. According to the Te−O vibration at 834 cm^−1^, it could be concluded that the Ni−O bonds in the [Ni(OTeF_5_)_4_]^2−^ anion have an ionic character. In fact, they are easily dissociated when the complex is dissolved in a donor solvent, as was proved by the formation of [Ni(NCMe)_6_][OTeF_5_]_2_ (**2**) in an acetonitrile solution. By slow gas diffusion of Et_2_O into a CH_2_Cl_2_ solution of **1**, dissociation of the teflate ligands can be prevented and two Et_2_O molecules coordinate in the axial positions of a distorted octahedron, whereas the teflate ligands are pushed into the equatorial plane resulting in [NEt_4_]_2_[*trans‐*Ni(OEt_2_)_2_(OTeF_5_)_4_] ⋅ CH_2_Cl_2_ (**3**). Unlike the unselective reaction of compound **1** with bpy, the reaction with the related bpyMe_2_ ligand yields the five‐coordinate [NEt_4_][Ni(bpyMe_2_)(OTeF_5_)_3_] species (**4**), in which only one teflate ligand has been displaced. The challenging chemistry of Ni(II) teflate species can now be better understood and paves the way to unveiling the viability of the [OTeF_5_]^−^ ligand to also stabilize the high oxidation state +IV of nickel. Additionally, the ligand‐field properties of the pentafluoorthotellurate group have been clarified and therefore the study of the magnetic and structural properties of different teflate coordination complexes can be tackled from a new perspective.

## Experimental Section

### General procedures and materials

All experiments were performed under rigorous exclusion of moisture and oxygen using standard Schlenk techniques. Solids were handled in a MBRAUN UNIlab plus glovebox under an argon atmosphere (O_2_<0.5 ppm, H_2_O<0.5 ppm). Solvents were dried using a MBraun SPS‐800 solvent system (CH_2_Cl_2_, CH_3_CN, *n*‐pentane), or with CaH_2_ (*o*DFB, Et_2_O, CD_3_CN) before use and stored over 3 or 4 Å molecular sieves. [NEt_4_]_2_[NiCl_4_]^[47]^ and ClOTeF_5_ ^[19b]^ were prepared as described elsewhere. 2,2’‐dimethyl‐6,6’‐bipyridine (bpyMe_2_) was dried at 120 °C under dynamic vacuum before use. All other reagents were purchased from standard commercial suppliers and used as received. Elemental analyses (CHNS) were carried out using a VARIO EL elemental analyzer. IR spectra were measured on neat solid samples at room temperature inside a glovebox under an argon atmosphere using a Bruker ALPHA FTIR spectrometer with a diamond ATR attachment with 32 scans and a resolution of 4 cm^−1^. NMR spectra were recorded on a JEOL 400 MHz ECS spectrometer. Crystal data were collected with MoKα radiation on a Bruker D8 Venture diffractometer with a CMOS area detector. Single crystals were picked at −40 °C under nitrogen atmosphere and mounted on a 0.15 mm Mitegen micromount using perfluoroether oil. The structures were solved with the ShelXT^[48]^ structure solution program using intrinsic phasing and refined with the ShelXL^[49]^ refinement package using least squares minimizations by using OLEX2.^[50]^ The program Diamond V4.6.4 was used for visualization.^[51]^ Crystal data and other details of the structure analyses are summarized in Tables S1–S4. Suitable crystals for X‐ray diffraction studies were obtained as indicated in the corresponding experimental entry.

### Synthesis of [NEt_4_]_2_[Ni(OTeF_5_)_4_] (1)

[NEt_4_]_2_[NiCl_4_] (100 mg, 217 μmol, 1 equiv.) was weighed into a Schlenk flask with a greaseless Teflon stopcock. After cooling down to −196 °C, ClOTeF_5_ (357 mg, 1.30 mmol, 6 equiv.) condensed onto it. The mixture was allowed to warm to room temperature under stirring, resulting in a brownish slurry, whereas evolution of a yellow gas was observed. The gas was identified as Cl_2_ by gas‐phase UV‐vis spectroscopy (see Figure S1). After 30 minutes of stirring, all volatiles were removed under vacuum. After drying overnight under vacuum a deep blue solid was obtained and identified as compound **1** (275 mg, 216 μmol, quant.). **IR** (ATR, 298 K, Figure S3): $\tilde{\nu }$ /cm^−1^=3001 (w), 1486 (w), 1459 (w), 1443 (w), 1397 (w), 1368 (w), 1186 (w), 1173 (w), 1069 (w), 1054 (w), 1032 (w), 834 (m, Te−O), 783 (m), 662 (vs, Te−F), 614 (w), 445 (m, Ni−O). **Elemental analysis** calcd (%) for C_16_H_40_F_20_N_2_NiO_4_Te_4_: C 15.1, H 3.2, N 2.2; found: C 15.4, H 3.2, N 2.2. **UV‐Vis** (CH_2_Cl_2_, Figure 3): *λ*_max_/nm=573, 630, 766, 1175.

### Crystal growth of [NEt_4_]_2_[Ni(OTeF_5_)_4_] (1)

Single crystals of **1** suitable for X‐ray diffraction were obtained by slow evaporation of a solution of the compound in *o*DFB (ca. 20 mg in 1 mL). This solution was placed in a Y‐shaped schlenk tube with a greaseless Teflon stopcock, and the empty side arm was placed in a cooling bath, which was cooled from room temperature to 0 °C along 15 h, controlled by a cryostat. Blue single crystals of [NEt_4_]_2_[Ni(OTeF_5_)_4_] (**1**) were obtained.

### Synthesis and crystal growth of [Ni(NCMe)_6_][OTeF_5_]_2_ (2)

[NEt_4_]_2_[Ni(OTeF_5_)_4_] (30 mg, 23.6 μmol) was dissolved in 1 mL MeCN in a Y‐shaped schlenk tube with a greaseless Teflon stopcock. In the side arm, 2 mL Et_2_O were added. After storing the tube at −24 °C for some days, pale purple single crystals suitable for X‐ray diffraction were obtained. Additionally, the supernatant solution could be removed with a syringe and after washing with Et_2_O (2×1 mL) and drying shortly under vacuum, compound **2** could be obtained as purple crystals (17 mg, 21.7 μmol, 92 % yield). **IR** (ATR, 298 K, Figure S4): $\tilde{\nu }$ /cm^−1^=3000 (w), 2943 (w), 2326 (w, *ν*_3_+*ν*_4_), 2299 (m, *ν*_2_: C≡N), 1419 (w), 1375 (w), 1039 (w), 944 (w), 865 (s, Te−O), 813 (m), 685 (m), 665 (m), 630 (vs, Te−F), 578 (w), 463 (w), 409 (m).

### Crystal growth of [NEt_4_]_2_[trans‐Ni(OEt_2_)_2_(OTeF_5_)_4_]⋅CH_2_Cl_2_ (3)

[NEt_4_]_2_[Ni(OTeF_5_)_4_] (10 mg, 7.9 μmol) was dissolved in 0.5 mL CH_2_Cl_2_ in a Y‐shaped schlenk tube with a greaseless Teflon stopcock. In the side arm, 1 mL Et_2_O was added. After storing the tube at −24 °C for some days, orange single crystals suitable for X‐ray diffraction were obtained.

### Synthesis of [NEt_4_][Ni(bpyMe_2_)(OTeF_5_)_3_] (4)

A mixture of [NEt_4_]_2_[Ni(OTeF_5_)_4_] (50 mg, 39.3 μmol, 1 equiv.) and bpyMe_2_ (7.2 mg, 39.3 μmol, 1 equiv.) was dissolved in *o*DFB (1 mL). The mixture turned green while undissolved material disappeared progressively. After stirring for 5 min, the solvent was evaporated until there was ca. 0.5 mL left. *n‐*pentane (0.5 mL) was added and a pale yellow‐green solid precipitated. The solvent was filtered off, and the resulting solid dried under vacuum. Some [NEt_4_][OTeF_5_] still remained in the sample. Keeping a solution of the obtained mixture in *o*DFB or CH_2_Cl_2_ (1 mL) at −24 °C for some days did not enable to completely remove it to obtain **4** as a pure substance. **IR** of the obtained solid material (ATR, 298 K, Figure S5): $\tilde{\nu }$ /cm^−1^=3000 (w), 1606 (w), 1574 (w), 1486 (m), 1474 (m), 1457 (m), 1443 (w), 1396 (w), 1368 (w), 1309 (w), 1262 (w), 1249 (w), 1183 (m), 1175 (w), 1132 (w), 1031 (w), 1025 (w), 1001 (w), 864 (m, Te−O, [OTeF_5_]^−^), 852 (s, Te−O), 845 (m), 792 (m), 784 (m), 656 (vs, Te−F), 634 (vs, Te−F, [OTeF_5_]^−^), 598 (m), 574 (w).

### Crystal growth of [NEt_4_][Ni(bpyMe_2_)(OTeF_5_)_3_] (4)

The mixture of [NEt_4_][Ni(bpyMe_2_)(OTeF_5_)_3_] and [NEt_4_][OTeF_5_] prepared as described previously was dissolved in 1 mL *o*DFB in a Y‐shaped schlenk tube with a greaseless Teflon stopcock. In the side arm, 1.5 mL *n‐*pentane were added. After storing the tube at −24 °C for some days, yellow single crystals suitable for X‐ray diffraction were obtained.

Deposition Numbers 2171818 (for **1**), 2172253 (for **2**), 2172098 (for **3**), 2172131 (for **4**) contain the supplementary crystallographic data for this paper. These data are provided free of charge by the joint Cambridge Crystallographic Data Centre and Fachinformationszentrum Karlsruhe Access Structures service.

## Conflict of interest

The authors declare no conflict of interest.

1

## Supporting information

As a service to our authors and readers, this journal provides supporting information supplied by the authors. Such materials are peer reviewed and may be re‐organized for online delivery, but are not copy‐edited or typeset. Technical support issues arising from supporting information (other than missing files) should be addressed to the authors.

Supporting Information

## Data Availability

The data that support the findings of this study are available from the corresponding author upon reasonable request.
