# Supplementary material for: Unravelling the Role of the Pentafluoroorthotellurate Group as a Ligand in Nickel Chemistry
Source: Chemistry. 2022 Sep 8;28(63):e202202016. doi: 10.1002/chem.202202016 (PMC9825845; doi:10.1002/chem.202202016)
Supplement: Supplementary file 1 — Supporting Information [file CHEM-28-0-s001.pdf]

Chemistry–A European Journal

Supporting Information

Unravelling the Role of the Pentafluoroorthotellurate Group as a Ligand in Nickel Chemistry

Alberto Pérez-Bitrián, Kurt F. Hoffmann, Konstantin B. Krause, Günther Thiele, Christian Limberg, and Sebastian Riedel*

Table of contents

1. Analysis of the gas phase formed in the synthesis of **1** S2
2. Behavior of $[NEt_4]_2[Ni(OTeF_5)_4]$ (**1**) in CD_3CN S3
3. IR spectra S4
4. Crystal data S6
5. Thermogravimetric analysis S14
6. Magnetic susceptibility measurements S15
7. Electronic spectrum analysis S20
8. References S21

1 Analysis of the gas phase formed in the synthesis of 1

The resulting gas phase, which was obtained in the synthesis of $[NEt_4]_2[Ni(OTeF_5)_4]$ (**1**, see Scheme 1), was analyzed by UV-Vis spectroscopy. It was transferred into a quartz cuvette and the gas-phase UV/Vis spectrum was recorded on a PerkinElmer Lambda 465. Elemental Cl_2, which was formed during the reaction, can be easily observed in the spectrum, together with the excess of unreacted $ClOTeF_5$ (see Figure S1).

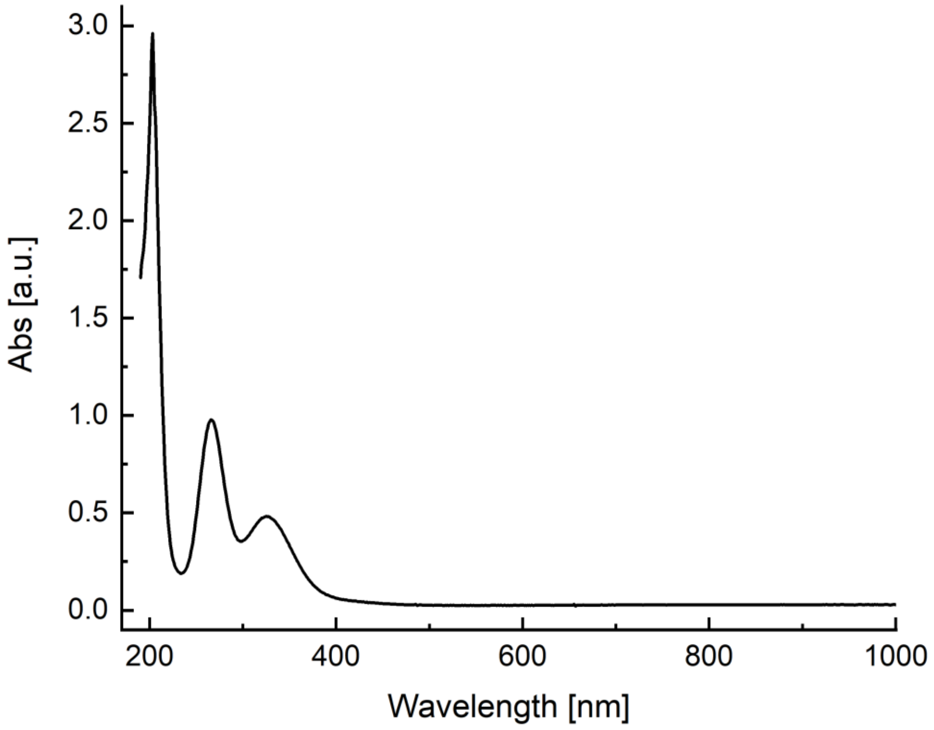

Figure S1. UV-Vis spectrum of the gas phase generated in the synthesis of $[NEt_4]_2[Ni(OTeF_5)_4]$ (**1**). The band at λ_{max} = 325 nm corresponds to Cl_2,[1] which is formed according to the reaction showed in Scheme 1. The band at λ_{max} = 266 nm corresponds to the excess of $ClOTeF_5$ used in such reaction.

2 Behavior of $[NEt_4]_2[Ni(OTeF_5)_4]$ (1) in CD_3CN

$[NEt_4]_2[Ni(OTeF_5)_4]$ (20 mg, 15.7 µmol) was dissolved in 0.6 mL CD_3CN. A pale blue solution was obtained. ^{19}F NMR data associated with the $[OTeF_5]^-$ anion in solution are in line with those found for the $[N^nBu_4]^+$ salt in CD_2Cl_2,[2] indicating dissociation of the pentafluoroorthotellurate ligand. The quality of the spectrum is rather poor due to the paramagnetism of the solvated Ni^{2+} cation (see Figure S2).

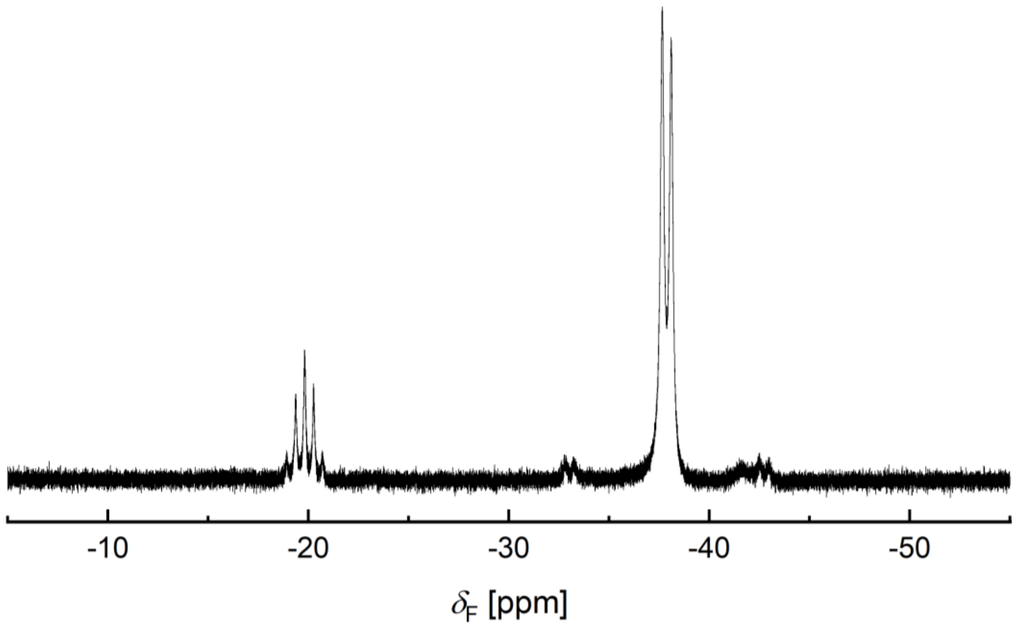

Figure S2. ^{19}F NMR spectrum (376 MHz) of the solution of compound $[NEt_4]_2[Ni(OTeF_5)_4]$ (**1**) in CD_3CN at 292 K. The signals show the typical AB_4 pattern of the pentafluoroorthotellurate group and correspond to the free $[OTeF_5]^-$ anion.[2] The ^{125}Te (I = 1/2) satellites in the low-field signal are not visible due to the poor quality of the spectrum arising from the paramagnetism of the solvated Ni^{2+} cation.

3 IR spectra

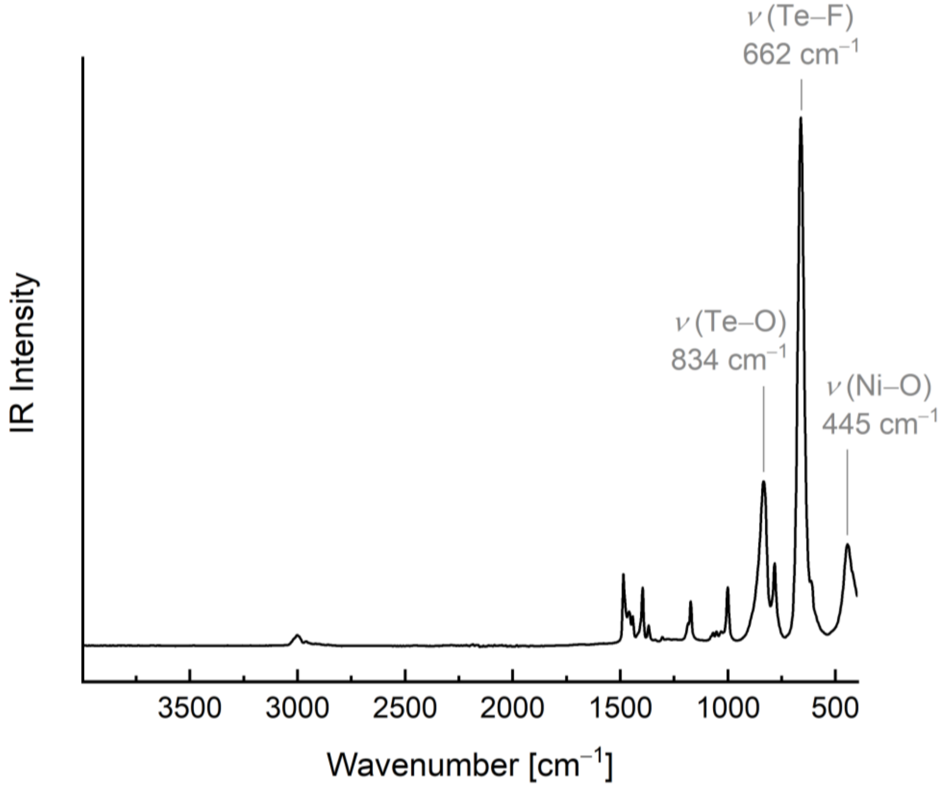

Figure S3. IR spectrum of compound $[NEt_4]_2[Ni(OTeF_5)_4]$ (**1**).

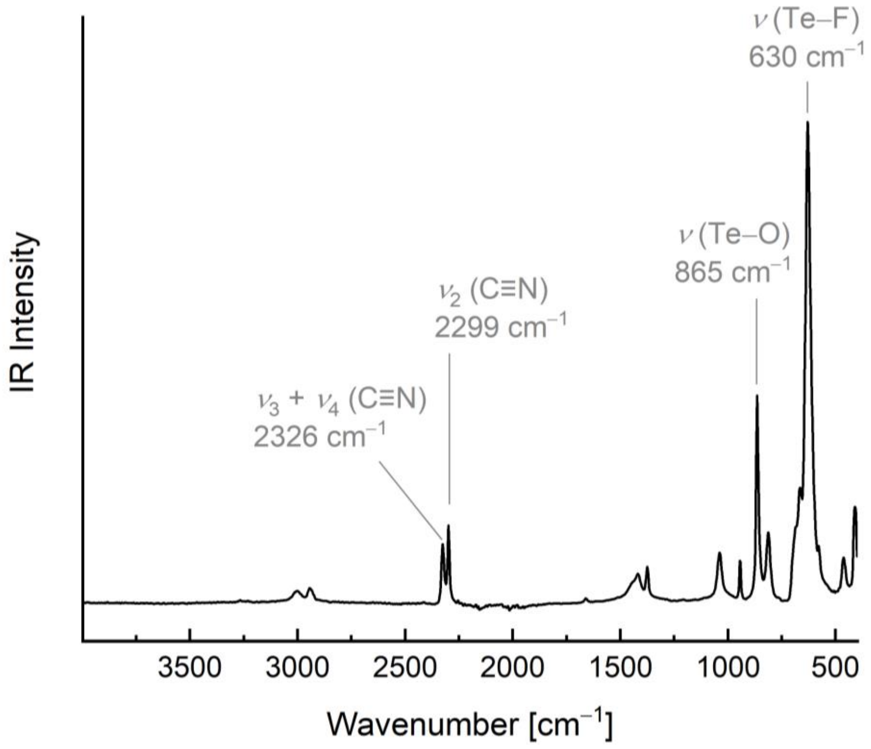

Figure S4. IR spectrum of compound $[Ni(NCMe)_6][OTeF_5]_2$ (**2**).

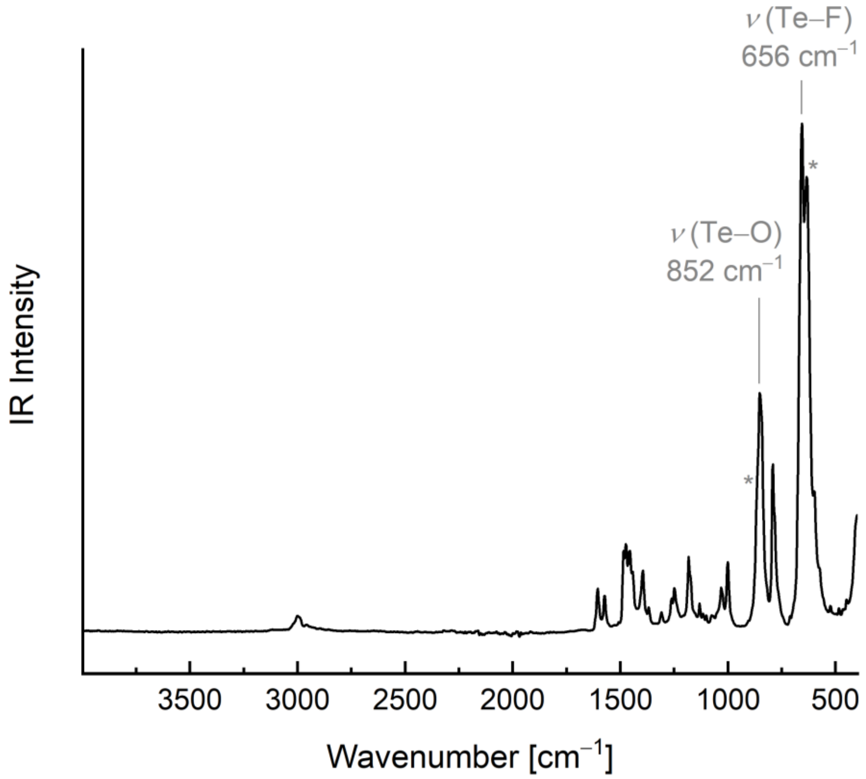

Figure S5. IR spectrum of the obtained mixture containing compound $[NEt_4][Ni(bpyMe_2)(OTeF_5)_3]$ (**4**). Marked signals correspond to free $[OTeF_5]^-$.[2]

4 Crystal data

4.1 Summary of crystal data and structure refinement

Table S1. Crystal data and structure refinement for compound **1**.

Empirical formula	$C_{16}H_{40}F_{20}N_2NiO_4Te_4$
Formula weight	1273.61
Temperature [K]	299.0
Crystal system	monoclinic
Space group	$P2_1/c$
a [pm]	1226.03(8)
b [pm]	1851.26(13)
c [pm]	1633.36(11)
α [°]	90
β [°]	95.514(2)
γ [°]	90
Volume [Å^3]	3690.1(4)
Z	4
ρ_{calc} [g·cm^{-3}]	2.292
μ [mm^{-1}]	3.753
F(000)	2392.0
Crystal size [mm^3]	0.61 × 0.197 × 0.058
Radiation	MoKα (λ = 0.71073)
2Θ range for data collection [°]	3.998 to 52.748
Index ranges	$-15 \leq h \leq 15$, $0 \leq k \leq 23$, $0 \leq l \leq 20$
Reflections collected	7711
Independent reflections	7711 [R_{int} = 0.0561, R_{sigma} = 0.0482]
Data/restraints/parameters	7711/0/432
Goodness-of-fit on F^2	1.189
Final *R* indexes [$I >= 2\sigma(I)$]	R_1 = 0.0491<br>wR_2 = 0.0843
Final *R* indexes [all data]	R_1 = 0.0626<br>wR_2 = 0.0893
Largest diff. peak/hole [e·Å^{-3}]	1.10/−1.20
CCDC number	2171818

Table S2. Crystal data and structure refinement for compound **2**.

Empirical formula	$C_{12}H_{18}F_{10}N_6NiTe_2O_2$
Formula weight	782.23
Temperature [K]	273.15
Crystal system	trigonal
Space group	*R*-3
a [pm]	1108.14(6)
b [pm]	1108.14(6)
c [pm]	1734.88(12)
α [°]	90
β [°]	90
γ [°]	120
Volume [Å^3]	1845.0(2)
Z	3
ρ_{calc} [g·cm–^{3}]	2.112
μ [mm^{-1}]	3.209
F(000)	1110.0
Crystal size [mm^3]	0.458 × 0.476 × 0.617
Radiation	MoKα (λ = 0.71073)
2Θ range for data collection [°]	4.85 to 58.312
Index ranges	$-15 \leq h \leq 15$, $-15 \leq k \leq 14$, $-23 \leq l \leq 23$
Reflections collected	6882
Independent reflections	1037 [R_{int} = 0.0397, R_{sigma} = 0.0324]
Data/restraints/parameters	1037/0/53
Goodness-of-fit on F^2	1.092
Final *R* indexes [$I >= 2\sigma (I)$]	R_1 = 0.0425, wR_2 = 0.1272
Final *R* indexes [all data]	R_1 = 0.0467, wR_2 = 0.1302
Largest diff. peak/hole [e·Å^{-3}]	1.74/−1.09
CCDC number	2172253

Table S3. Crystal data and structure refinement for compound **3**.

Empirical formula	$C_{25}H_{62}Cl_2F_{20}N_2NiO_6Te_4$
Formula weight	1506.77
Temperature [K]	273.15
Crystal system	orthorhombic
Space group	*Pcca*
a [pm]	3181.88(19)
b [pm]	1428.28(9)
c [pm]	2111.97(14)
α [°]	90
β [°]	90
γ [°]	90
Volume [Å^3]	9598.1(10)
Z	8
ρ_{calc} [g·cm–^{3}]	2.085
μ [mm^{-1}]	3.014
F(000)	5792.0
Crystal size [mm^3]	0.2 × 0.169 × 0.095
Radiation	MoKα (λ = 0.71073)
2Θ range for data collection [°]	3.832 to 52.766
Index ranges	$-39 \leq h \leq 39$, $-17 \leq k \leq 17$, $-26 \leq l \leq 26$
Reflections collected	378730
Independent reflections	9827 [R_{int} = 0.0587, R_{sigma} = 0.0139]
Data/restraints/parameters	9827/680/631
Goodness-of-fit on F^2	1.158
Final *R* indexes [$I >= 2\sigma (I)$]	R_1 = 0.0201, wR_2 = 0.0418
Final *R* indexes [all data]	R_1 = 0.0237, wR_2 = 0.0430
Largest diff. peak/hole [e·Å^{-3}]	0.45/−0.89
CCDC number	2172098

Table S4. Crystal data and structure refinement for compound **4**.

Empirical formula	$C_{20}H_{32}F_{15}N_3NiO_3Te_3$
Formula weight	1088.99
Temperature [K]	100.0
Crystal system	triclinic
Space group	*P*-1
a [pm]	1513.3(2)
b [pm]	1520.7(2)
c [pm]	1553.0(2)
α [°]	76.641(5)
β [°]	74.644(5)
γ [°]	75.753(5)
Volume [$Å^3$]	3287.3(8)
Z	4
ρ_{calc} [g·cm–^{3}]	2.200
μ [mm^{-1}]	3.313
F(000)	2064.0
Crystal size [mm^3]	0.595 × 0.379 × 0.362
Radiation	MoKα (λ = 0.71073)
2Θ range for data collection [°]	4.276 to 52.9
Index ranges	$-17 \leq h \leq 18$, $-18 \leq k \leq 19$, $0 \leq l \leq 19$
Reflections collected	24487
Independent reflections	24487 [R_{int} = 0.0336, R_{sigma} = 0.0220]
Data/restraints/parameters	24487/0/823
Goodness-of-fit on F^2	1.055
Final *R* indexes [$I >= 2\sigma (I)$]	R_1 = 0.0372, wR_2 = 0.0934
Final *R* indexes [all data]	R_1 = 0.0401, wR_2 = 0.0960
Largest diff. peak/hole [e·$Å^{-3}$]	1.14/−1.20
CCDC number	2172131

4.2 Molecular structure of $[NEt_4]_2[Ni(OTeF_5)_4]$ (**1**) in the solid state

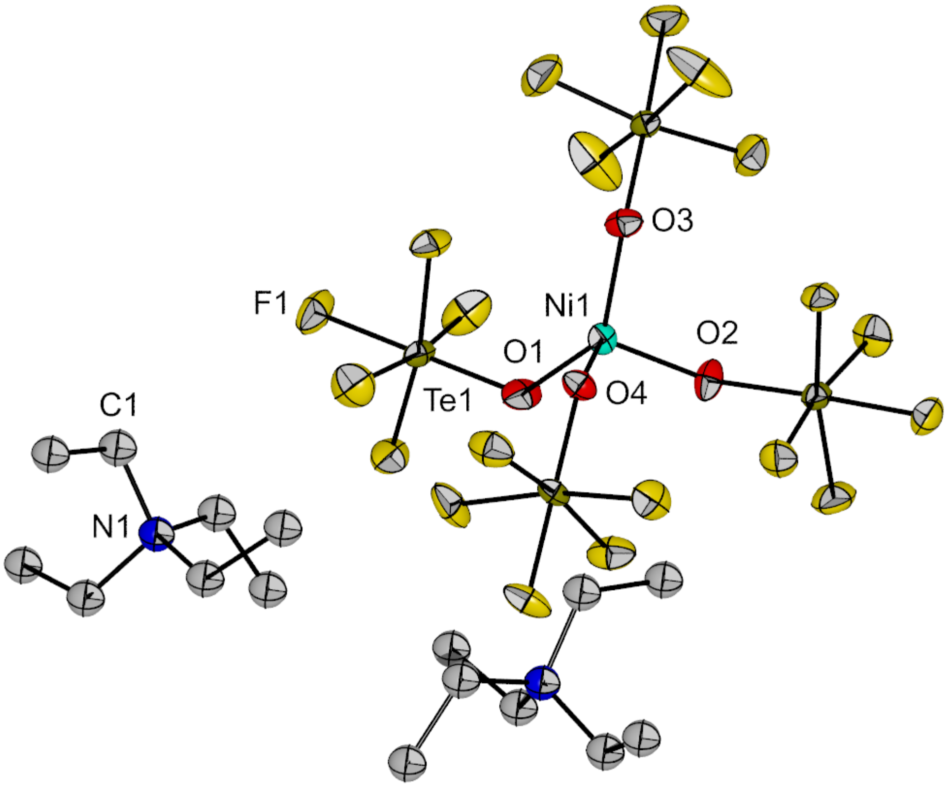

Figure S6. Molecular structure of $[NEt_4]_2[Ni(OTeF_5)_4]$ (**1**) in the solid state. Hydrogen atoms have been omitted for clarity. Displacement ellipsoids set at 50% probability.

4.3 Solid-state structure of [Ni(NCMe)$_6$][OTeF$_5$]$_2$ (**2**)

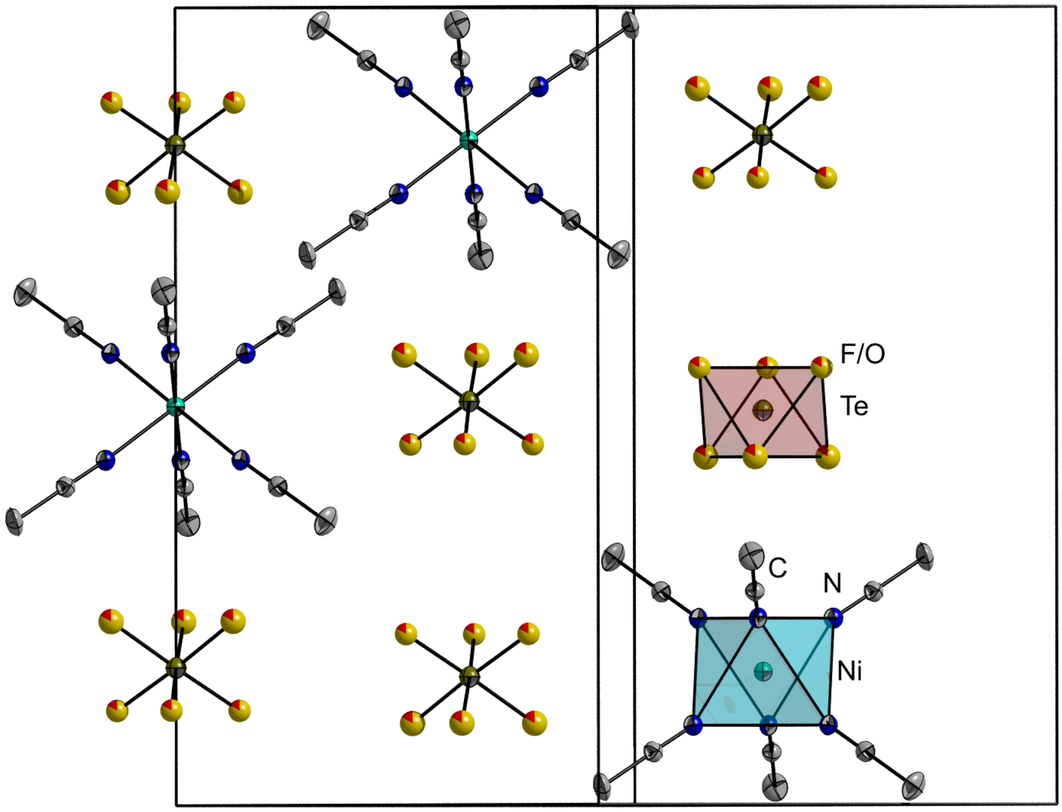

Figure S7. Solid-state structure of [Ni(NCMe)$_6$][OTeF$_5$]$_2$ (**2**). Hydrogen atoms have been omitted for clarity. Displacement ellipsoids set at 50% probability. Coordination octahedra are shown for both the cation and the anion.

4.4 Molecular structure of $[NEt_4]_2[\textit{trans}\text{-}Ni(OEt_2)_2(OTeF_5)_4]\cdot CH_2Cl_2$ (**3**) in the solid state

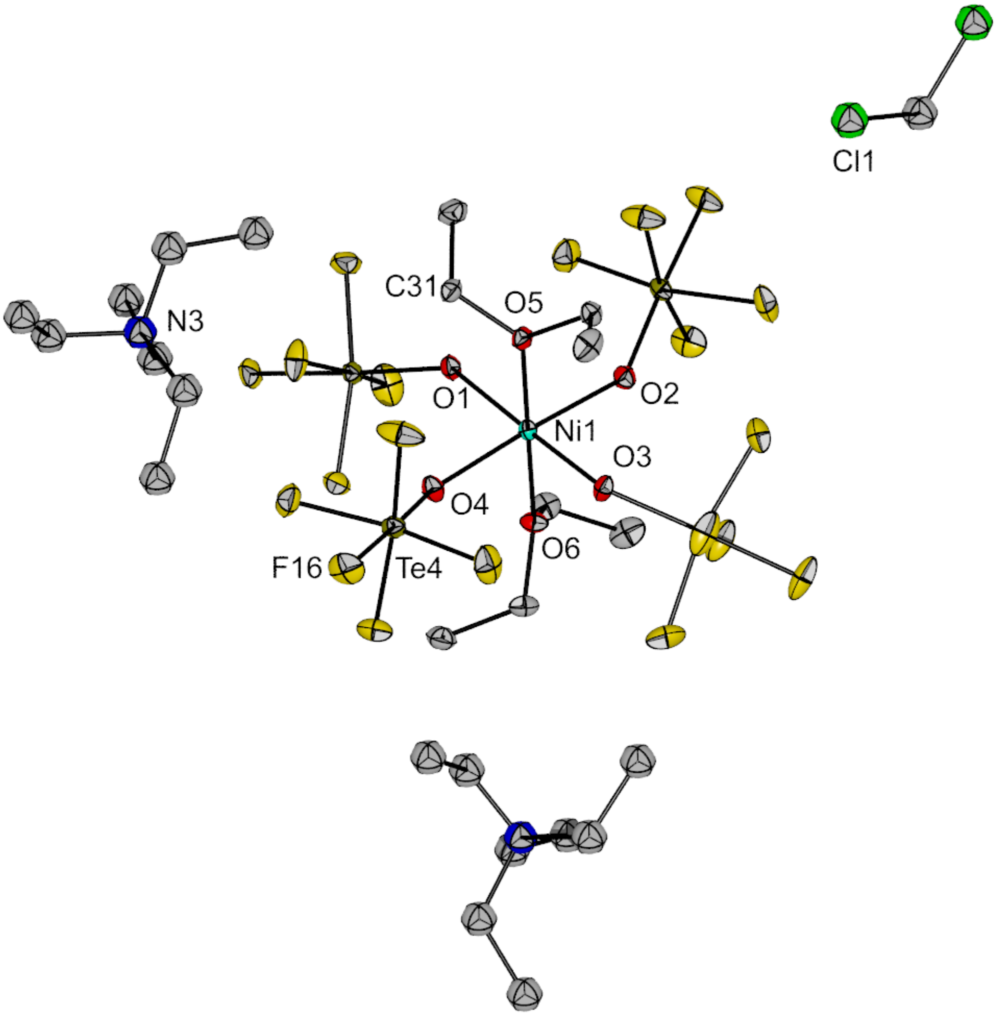

Figure S8. Molecular structure of $[NEt_4]_2[\textit{trans}\text{-}Ni(OEt_2)_2(OTeF_5)_4]\cdot CH_2Cl_2$ (**3**) in the solid state. Hydrogen atoms have been omitted for clarity. Displacement ellipsoids set at 50% probability. Both $[NEt_4]^+$ cations are half occupied and only one set of each is shown.

4.5 Molecular structure of $[NEt_4][Ni(bpyMe_2)(OTeF_5)_3]$ (**4**) in the solid state

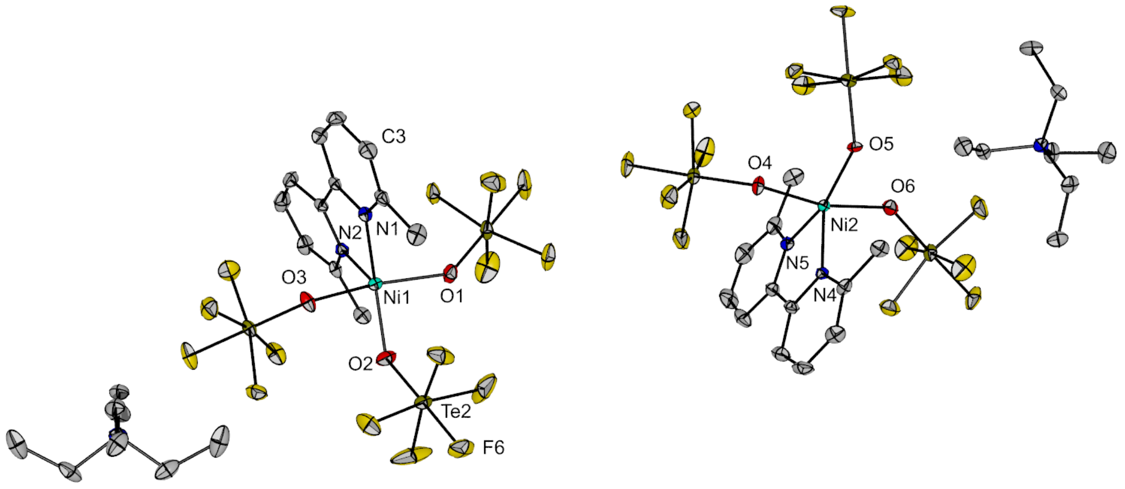

Figure S9. Molecular structure of $[NEt_4][Ni(bpyMe_2)(OTeF_5)_3]$ (**4**) in the solid state. Hydrogen atoms have been omitted for clarity. Displacement ellipsoids set at 50% probability.

5 Thermogravimetric analysis

The thermogravimetric analysis (TGA) of $[NEt_4]_2[Ni(OTeF_5)_4]$ (**1**) was performed using a STA 449 F3 JUPITER instrument at a heating rate of 10 °C min^{-1} under an argon atmosphere. The spectrum was processed using NETZSCH Proteus (see Figure S10).

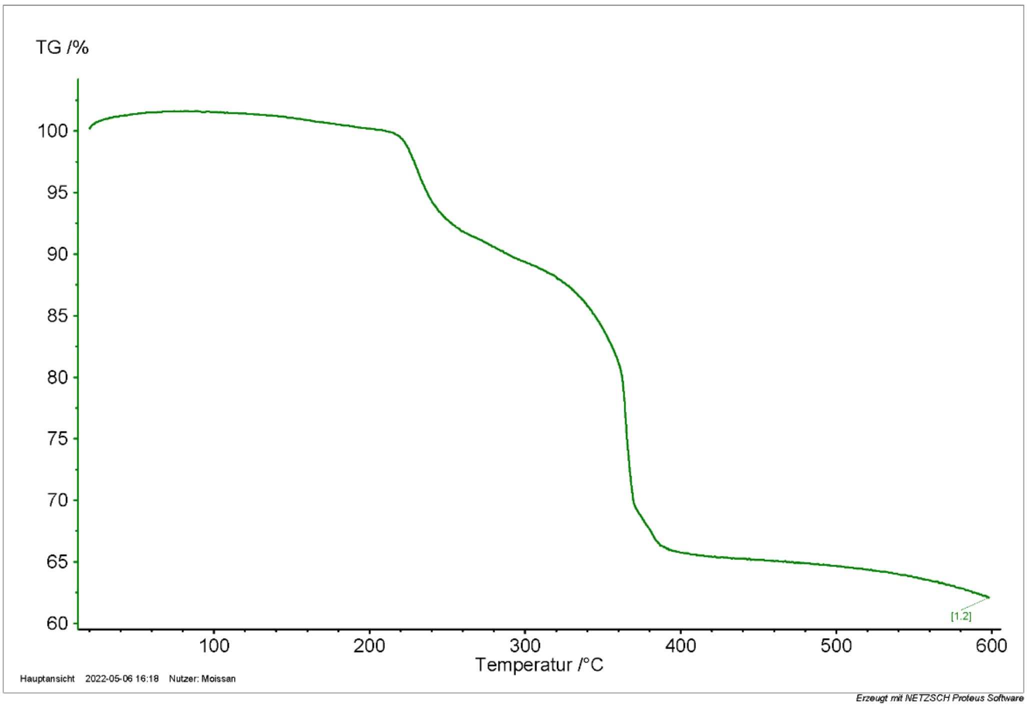

Figure S10. TGA of compound $[NEt_4]_2[Ni(OTeF_5)_4]$ (**1**) recorded on heating at 10 °C min^{-1}.

6 Magnetic susceptibility measurements

6.1 Superconducting quantum interference device (SQUID)

Magnetic measurements were performed with a QuantumDesign MPMS3 SQUID magnetometer. The samples were prepared in a glove box in VSM powder capsules, which were previously dried at 110 °C under vacuum for five days. A brass sample holder was used. The measurement was carried out in VSM mode from 2 K to 300 K in a magnetic field of 7 T after cooling down at zero field. A background correction was applied by subtracting the magnetic moment of an empty capsule, determined by using the same measurement sequence as for the sample. A diamagnetic correction of the molar susceptibilities was performed using Pascal's constants.[3] The experimental magnetic data are collected in Table S5, and the plot of μ_{eff} versus T for complex $[NEt_4]_2[Ni(OTeF_5)_4]$ (**1**) is shown in Figure 2.

Table S5. Experimental magnetic data for $[NEt_4]_2[Ni(OTeF_5)_4]$ (**1**) obtained from the SQUID measurement.[a]

***T* [K]**	**Magnetic Field [Oe]**	**Magnetic Moment [emu]**	χ_{mol} **[cm³ mol⁻¹]**	χ_p[b] **[cm³ mol⁻¹]**	μ_{eff} **[μB]**
2.000	69999.54	0.03497	0.06300	0.06353	1.0079
2.104	69999.54	0.03497	0.06300	0.06353	1.0340
2.213	69999.54	0.03485	0.06277	0.06330	1.0585
2.328	69999.54	0.03494	0.06293	0.06346	1.0870
2.449	69999.54	0.03488	0.06283	0.06336	1.1139
2.576	69999.54	0.03493	0.06293	0.06346	1.1433
2.709	69999.54	0.03497	0.06299	0.06352	1.1731
2.850	69999.54	0.03489	0.06285	0.06338	1.2020
2.998	69999.54	0.03491	0.06289	0.06342	1.2331
3.154	69999.54	0.03492	0.06290	0.06343	1.2649
3.318	69999.54	0.03491	0.06289	0.06342	1.2972
3.490	69999.54	0.03479	0.06268	0.06321	1.3282
3.671	69999.54	0.03483	0.06274	0.06327	1.3630
3.862	69999.54	0.03480	0.06269	0.06322	1.3973
4.063	69999.54	0.03476	0.06262	0.06315	1.4324
4.274	69999.54	0.03475	0.06260	0.06313	1.4689
4.496	69999.54	0.03480	0.06269	0.06322	1.5077

4.729	69999.54	0.03477	0.06264	0.06317	1.5457
4.974	69999.54	0.03473	0.06256	0.06309	1.5842
5.232	69999.54	0.03462	0.06236	0.06289	1.6221
5.504	69999.54	0.03453	0.06221	0.06274	1.6617
5.790	69999.54	0.03453	0.06221	0.06274	1.7045
6.091	69999.54	0.03453	0.06220	0.06273	1.7481
6.406	69999.54	0.03447	0.06209	0.06262	1.7911
6.739	69999.54	0.03444	0.06204	0.06257	1.8364
7.089	69999.54	0.03426	0.06171	0.06224	1.8785
7.455	69999.54	0.03425	0.06170	0.06223	1.9263
7.843	69999.54	0.03410	0.06143	0.06196	1.9715
8.251	69999.54	0.03407	0.06136	0.06189	2.0209
8.679	69999.54	0.03398	0.06121	0.06174	2.0702
9.130	69999.54	0.03378	0.06085	0.06138	2.1170
9.604	69999.54	0.03365	0.06062	0.06115	2.1671
10.102	69999.54	0.03353	0.06039	0.06092	2.2186
10.627	69999.54	0.03330	0.05999	0.06052	2.2680
11.178	69999.54	0.03306	0.05955	0.06008	2.3175
11.759	69999.54	0.03289	0.05924	0.05977	2.3708
12.369	69999.54	0.03260	0.05873	0.05926	2.4212
13.012	69999.54	0.03236	0.05830	0.05883	2.4742
13.687	69999.54	0.03208	0.05779	0.05832	2.5267
14.398	69999.54	0.03171	0.05712	0.05765	2.5766
15.145	69999.54	0.03141	0.05658	0.05711	2.6300
15.931	69999.54	0.03099	0.05582	0.05635	2.6796
16.759	69999.54	0.03059	0.05511	0.05564	2.7308
17.628	69999.54	0.03017	0.05434	0.05487	2.7814
18.543	69999.54	0.02973	0.05355	0.05408	2.8320
19.506	69999.54	0.02921	0.05262	0.05315	2.8794
20.518	69999.54	0.02867	0.05164	0.05217	2.9259
21.584	69999.54	0.02822	0.05083	0.05136	2.9776
22.704	69999.54	0.02769	0.04987	0.05040	3.0252
23.883	69999.54	0.02706	0.04874	0.04927	3.0677
25.123	69999.54	0.02649	0.04772	0.04825	3.1135
26.427	69999.54	0.02585	0.04657	0.04710	3.1551

27.799	69999.54	0.02519	0.04537	0.04590	3.1946
29.242	69999.54	0.02458	0.04428	0.04481	3.2374
30.760	69999.54	0.02392	0.04310	0.04363	3.2761
32.357	69999.54	0.02327	0.04192	0.04245	3.3146
34.037	69999.54	0.02259	0.04070	0.04123	3.3501
35.804	69999.54	0.02191	0.03946	0.03999	3.3841
37.664	69999.54	0.02121	0.03820	0.03873	3.4158
39.617	69999.54	0.02058	0.03707	0.03760	3.4515
41.674	69999.54	0.01983	0.03573	0.03626	3.4762
43.837	69999.54	0.01916	0.03451	0.03504	3.5049
46.112	69999.54	0.01850	0.03333	0.03386	3.5335
48.507	69999.54	0.01779	0.03205	0.03258	3.5552
51.023	69999.54	0.01714	0.03088	0.03141	3.5799
53.670	69999.54	0.01648	0.02968	0.03021	3.6011
56.456	69999.54	0.01577	0.02840	0.02893	3.6144
59.385	69999.54	0.01513	0.02725	0.02778	3.6321
62.469	69999.54	0.01446	0.02605	0.02658	3.6442
65.712	69999.54	0.01381	0.02487	0.02540	3.6535
69.120	69999.54	0.01320	0.02377	0.02430	3.6650
72.705	69999.54	0.01261	0.02272	0.02325	3.6765
76.477	69999.54	0.01209	0.02178	0.02231	3.6938
80.446	69999.54	0.01154	0.02080	0.02133	3.7041
84.622	69999.54	0.01101	0.01984	0.02037	3.7129
89.011	69999.54	0.01048	0.01887	0.01940	3.7165
93.629	69999.54	0.01000	0.01802	0.01855	3.7269
98.486	69999.54	0.00956	0.01723	0.01776	3.7398
103.599	69999.54	0.00908	0.01636	0.01689	3.7407
108.983	69999.54	0.00865	0.01558	0.01611	3.7474
114.633	69999.54	0.00825	0.01487	0.01540	3.7574
120.587	69999.54	0.00788	0.01420	0.01473	3.7686
126.846	69999.54	0.00747	0.01346	0.01399	3.7668
133.432	69999.54	0.00713	0.01284	0.01337	3.7768
140.356	69999.54	0.00680	0.01224	0.01277	3.7865
147.646	69999.54	0.00646	0.01163	0.01216	3.7900
155.313	69999.54	0.00617	0.01112	0.01165	3.8033

163.373	69999.54	0.00586	0.01056	0.01109	3.8062
171.859	69999.54	0.00557	0.01003	0.01056	3.8089
180.779	69999.54	0.00528	0.00951	0.01004	3.8099
190.173	69999.54	0.00508	0.00914	0.00967	3.8356
200.044	69999.54	0.00483	0.00870	0.00923	3.8419
210.435	69999.54	0.00459	0.00826	0.00879	3.8464
221.358	69999.54	0.00439	0.00790	0.00843	3.8633
232.856	69999.54	0.00418	0.00752	0.00805	3.8727
244.947	69999.54	0.00398	0.00716	0.00769	3.8820
257.671	69999.54	0.00380	0.00684	0.00737	3.8977
271.058	69999.54	0.00361	0.00651	0.00704	3.9068
285.131	69999.54	0.00345	0.00622	0.00675	3.9237
299.932	69999.54	0.00327	0.00588	0.00641	3.9218

[a] m = 10.1 mg [b] χ_p = molar magnetic susceptibility after correction for the diamagnetism of the sample (χ_{dia} = –529.94·10^{-6} cm^3 mol^{-1}, calculated according to Ref. [3])

6.2 Magnetic susceptibility balance

The magnetic susceptibility was furthermore determined using an Alfa MSB-1 magnetic susceptibility balance. A sample tube with an inner diameter of 2 mm was used. The sample was prepared in the glovebox and the tube was sealed with silicon grease. A diamagnetic correction was applied using Pascal's constants.[3] Measured data can be found in Table S6 and the parameters calculated therefrom in Table S7.

The mass and molar susceptibilities were calculated using the data collected in Table S6 by means of the following equations:

$$\chi_g = \frac{l \cdot C \cdot (R - R_0)}{m}$$

$$\chi_{mol} = \chi_g \cdot MW$$

where MW = the molecular weight of the substance (1273.61 g mol^{-1} for $[NEt_4]_2[Ni(OTeF_5)_4]$, **1**).

A diamagnetic correction was applied to the molar magnetic susceptibility by using Pascal's constants (see Table S5)[3] and the following equation:

$$\chi_p = \chi_{mol} - \chi_{dia}$$

Finally, the effective magnetic moment was calculated by means of the equation that relates it with the molar magnetic susceptibility:

$$\mu_{eff} = 2.828\sqrt{\chi_p \cdot T}$$

Table S6. Measured data needed for the determination of the effective magnetic moment of $[NEt_4]_2[Ni(OTeF_5)_4]$ (**1**) using the magnetic susceptibility balance.

**Parameter**	**Value**	**Explanation**
m [mg]	49.5	Sample mass
l [cm]	1.65	Sample length
T [K]	295.65	Temperature
C	10^{-9}	Balance constant
R_0	−66	Empty reading
R	52	Sample reading

Table S7. Determined magnetic parameters for $[NEt_4]_2[Ni(OTeF_5)_4]$ (**1**).

**Parameter**	**Value**	**Explanation**
χ_g [cm^3 g^{-1}]	$3.93 \cdot 10^{-6}$	Mass susceptibility
χ_{mol} [cm^3 mol^{-1}]	$5.01 \cdot 10^{-3}$	Molar susceptibility
χ_p [cm^3 mol^{-1}]	$5.54 \cdot 10^{-3}$	Molar magnetic susceptibility after the diamagnetic correction
μ_{eff} [μ_B]	3.62	Effective magnetic moment

7 Electronic Spectrum Analysis

The electronic spectrum shown in Figure 3 was collected in CH_2Cl_2 solution (~0.01 M) in a Cary5000 UV-Vis-NIR from Agilent between 200 and 1600 nm. Further parameters associated with the measurement were: slit 2 nm, step 1 nm, grating change 800 nm, lamp change 350 nm.

The D_q and B parameters were calculated from the equations provided by Dou,[4] as graphical methods are usually less accurate. Due to the nature of $[NEt_4]_2[Ni(OTeF_5)_4]$ (**1**), which is a (distorted) tetrahedral d^8 species, the nickel center has a T_1 ground state. Only ν_2 and ν_3 are observed in our spectrum, as the position of ν_1 should appear at higher energies. Therefore, the ligand field and the Racah parameters can be calculated by means of the equations:

$$Dq = \left\{[85\nu_3^2 - 4(\nu_3 - 2\nu_2)^2]^{1/2} - 9(\nu_3 - 2\nu_2)\right\}/340$$

$$B = (\nu_3 - 2\nu_2 + 30Dq)/15$$

The value used for ν_3 was estimated to be the average of the two components observed for such absorption. For this work, we recalculated the Dq and B parameters of other tetrahedral Ni(II) species by taking the experimental values of ν_2 and ν_3 reported previously in the literature, to allow for a better comparison (see Table 1).

8 References

[1] D. Maric, J. P. Burrows, R. Meller, G. K. Moortgat, J. *Photochem. Photobiol. A* **1993**, *70*, 205.

[2] S. H. Strauss, K. D. Abney, O. P. Anderson, *Inorg. Chem.* **1986**, *25*, 2806.

[3] G. A. Bain, J. F. Berry, *J. Chem. Educ.* **2008**, *85*, 532.

[4] Y.-s. Dou, *J. Chem. Educ.* **1990**, *67*, 134.
